# Age Dependent Modification of the Metabolic Profile of the Tibialis Anterior Muscle Fibers in C57BL/6J Mice

**DOI:** 10.3390/ijms21113923

**Published:** 2020-05-30

**Authors:** Emiliana Giacomello, Emanuela Crea, Lucio Torelli, Alberta Bergamo, Carlo Reggiani, Gianni Sava, Luana Toniolo

**Affiliations:** 1 Department of Medicine, Surgery and Health Sciences, University of Trieste, 34149 Trieste, Italy; EMANUELA.CREA@studenti.units.it (E.C.); torelli@units.it (L.T.); 2 Department of Life Sciences, University of Trieste, Trieste, Italy, and Callerio Foundation, Onlus, 34127 Trieste, Italy; abergamo@units.it (A.B.); gsava@units.it (G.S.); 3 Laboratory of Muscle Biophysics, Department of Biomedical Sciences, University of Padova, 35131 Padova, Italy; carlo.reggiani@unipd.it

**Keywords:** skeletal muscle aging, muscle fiber, Myosin Heavy Chain, metabolic profile, capillaries

## Abstract

Skeletal muscle aging is accompanied by mass reduction and functional decline, as a result of multiple factors, such as protein expression, morphology of organelles, metabolic equilibria, and neural communication. Skeletal muscles are formed by multiple fibers that express different Myosin Heavy Chains (MyHCs) and have different metabolic properties and different blood supply, with the purpose to adapt their contraction to the functional need. The fine interplay between the different fibers composing a muscle and its architectural organization determine its functional properties. Immunohistochemical and histochemical analyses of the skeletal muscle tissue, besides evidencing morphological characteristics, allow for the precise determination of protein expression and metabolic properties, providing essential information at the single-fiber level. Aiming to gain further knowledge on the influence of aging on skeletal muscles, we investigated the expression of the MyHCs, the Succinate Dehydrogenase (SDH) activity, and the presence of capillaries and Tubular Aggregates (TAs) in the tibialis anterior muscles of physiologically aging C57BL/6J mice aged 8 (adult), 18 (middle aged), and 24 months (old). We observed an increase of type-IIB fast-contracting fibers, an increase of the oxidative capacity of type-IIX and -IIA fibers, a general decrease of the capillarization, and the onset of TAs in type-IIB fibers. These data suggest that aging entails a selective modification of the muscle fiber profiles.

## 1. Introduction

The contractile properties of a muscle are the result of finely coordinated functional properties of the different cells of which it is composed. In a healthy adult individual, the regulation of muscle contraction is achieved thanks to the orchestration of the heterogeneity of muscle fibers and their characteristics that, in turn, can vary depending the functional need, the onset of distinct pathologies, and the age. Muscles fibers differ from one another in the expression of distinct contractile apparatus and sarcoplasmic reticulum proteins; they have different Ca^2+^ handling properties and metabolic profiles (oxidative or glycolytic), and consequently have different contractile characteristics (fast- or slow-contracting) [1]. In this context, the Myosin Heavy Chain (MyHC) isoforms expressed in a muscle fiber are the main markers of its contractile and metabolic properties [2,3]. However, recent observational evidence demonstrates that muscles do not simply have the ability to modify the fiber type composition, but are also able to fine-tune the aerobic capacity, in order to fulfil the functional needs [4].

Aging is a biological process which encompasses modifications at cellular, the organ, and the system level. As with other tissues and organs, muscle undergoes age-dependent alterations, resulting not only in an impaired contractile function, but also in a perturbed general health condition. Skeletal muscle aging entails mass and power reduction, together with a decline of the resistance to fatigue, factors that are ascribable to the modification of multiple factors as contractile proteins, excitation–contraction components, neuromuscular junction, blood flow, metabolic properties, and atrophy mechanisms [5,6]. Skeletal muscle aging includes macroscopic effects, such as the change of the muscle architecture [7], and modifications at the cellular level, which involve the alteration of the morphology, fiber-type shift [8], change in the excitation–contraction coupling proteins [9], capillaries network [10], and the onset of tubular aggregates (TAs), as a sign of skeletal muscle damage [11,12,13], among the others. Although the cooperation of these factors provides an explanation of the effects of skeletal muscle aging, the mechanisms are still not completely understood. The idea that skeletal muscle function declines due to an overall skeletal muscle architecture modification, or a loss of the fine interplay that characterizes muscle contraction, rather than a change in single fiber parameters [7,14], guided our interest toward further elucidation of the properties of skeletal muscle during the aging progression.

In this context, immunohistochemical and histochemical analyses of the skeletal muscle tissue allow for both the precise determination of protein expression and metabolic properties at the single-fiber level and provide important information on the overall structure of the tissue. These two experimental determinants can lead to an underestimation of the changes if batch analyses are performed or there is information loss when isolated single-fiber properties are measured. 

The study of skeletal muscle aging in humans is complex, subject to high variability and to the influence of multiple factors, and, not least, introduces ethical concerns. Therefore, although the scientific community has recently become more aware that physiologically aging mice models do not fully mirror the human physiology of aging [6], studies on aging biology entail the use of the mice strains due to their versatility, possibility to have genetically modified models, and the possibility to perform highly reliable cross-sectional studies. 

In order to elucidate whether aging entails modification of structural, immunohistochemical, and metabolic properties, we performed a study on physiologically aging C57BL/6J mice, which entailed the analyses of the expression of the MyHC isoforms, the presence of capillaries, Succinate Dehydrogenase (SDH) activity, and presence of TAs to define the fiber profile of the fast-contracting muscle tibialis anterior of adult, middle-aged, and old mice [15,16]. Our data show that aging tibialis anterior muscle undergoes specific modifications, such as the increase of type-IIB fibers, the increase of SDH activity in a fiber-type specific manner, and the onset of TAs in type-IIB fibers, leading to suggest that the decline in skeletal muscle performance upon age progression is a multifactorial process. 

## 2. Results

### 2.1. Fiber-Type Composition in Aging Tibialis Anterior Muscle

**Figure 1:**
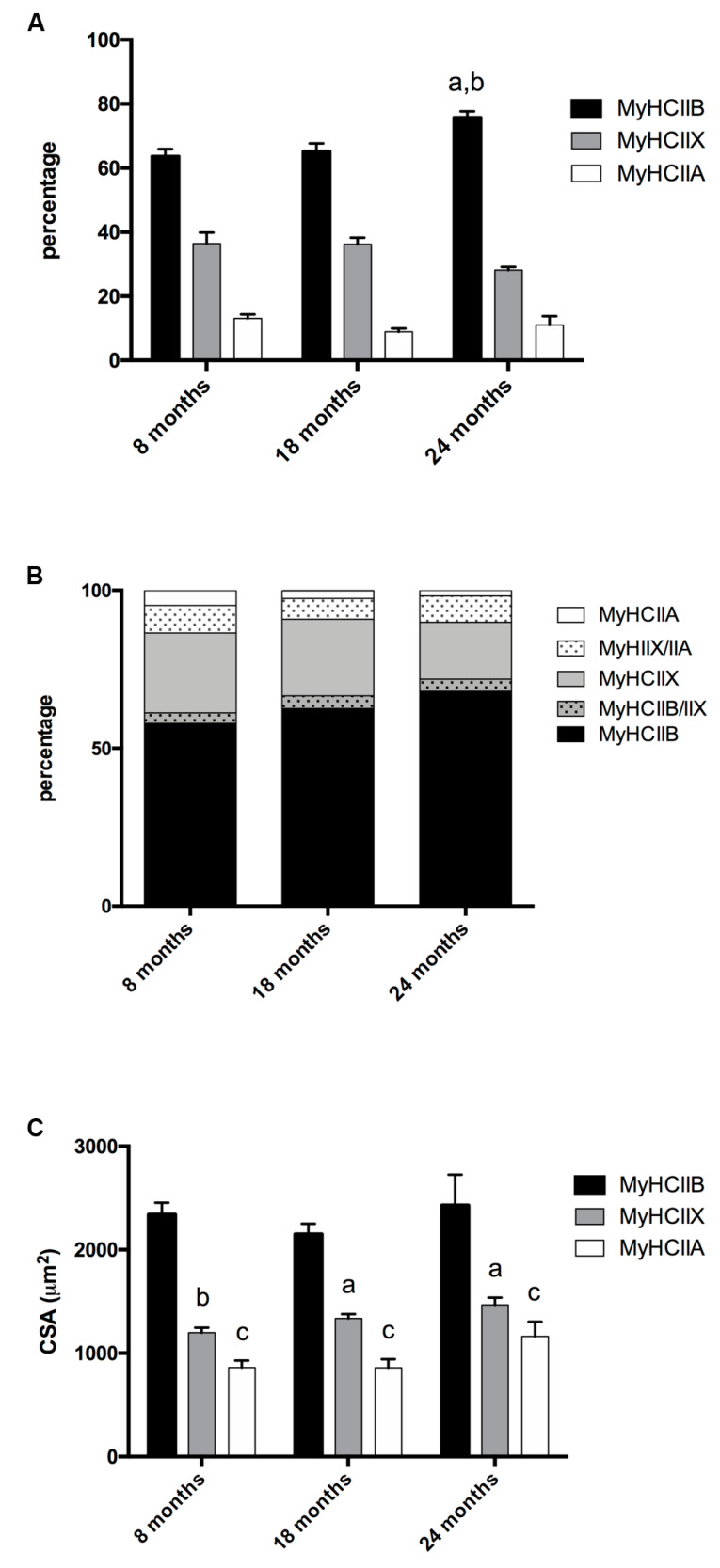
Fiber-type composition and fiber CSA of tibialis anterior during age progression. (**A**) Fiber-type composition varies during age progression, the percentage of IIB fibers increases significantly in 24-month-old mice (a, significantly different from eight-month-old mice, *p* < 0.05; b, significantly different from 18-month-old mice, *p* < 0.05). (**B**) Percentage of pure and hybrid fibers during age progression. (**C**) The CSA is fiber-type-dependent and does not display major modifications during age progression (a, significantly different from age-matched type-IIB fibers, *p* < 0.01; b, significantly different from age matched type-IIB fibers, *p* < 0.001; c, significantly different from age matched type-IIB fibers, *p* < 0.0001). Data are reported as means ± SEM.

**Figure 2:**
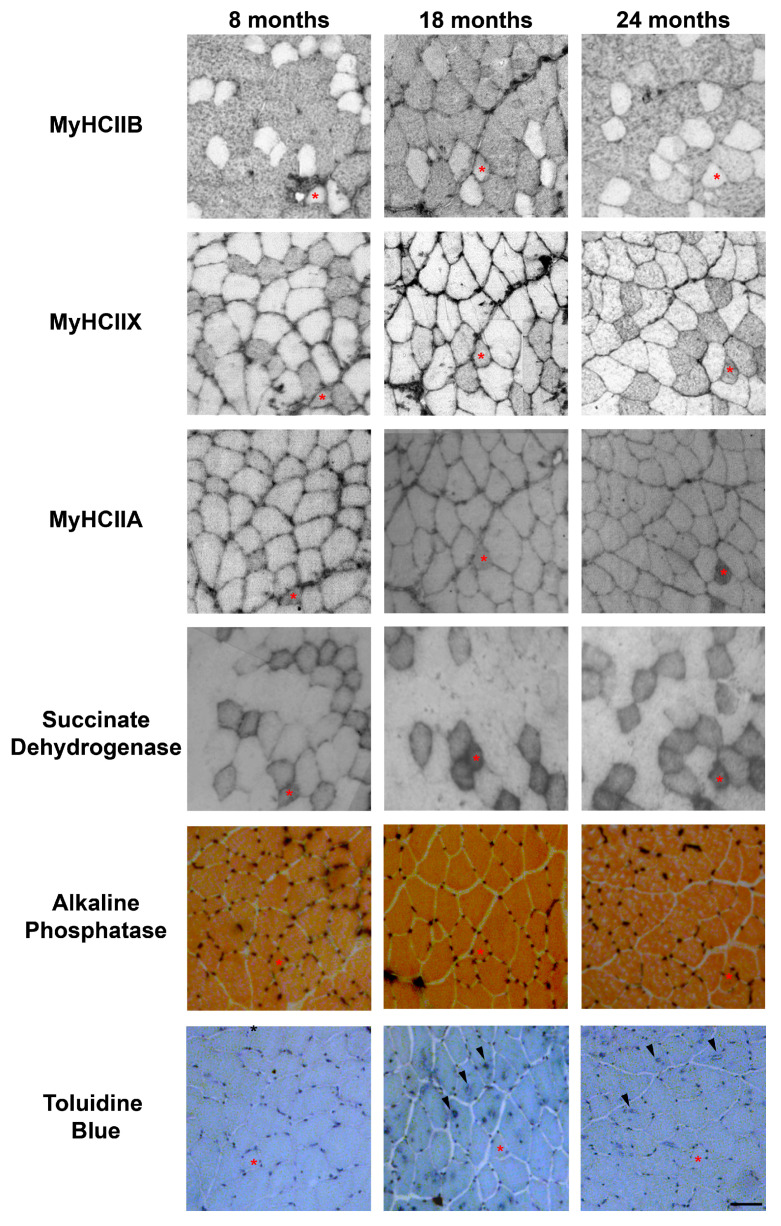
Representative examples of serial sections from aging tibialis anterior muscles, stained for MyHC IIB, IIX and IIA, succinate dehydrogenase, alkaline phosphatase to detect capillaries, and Toluidine Blue to evidence TAs. An asterisk indicates a IIX/IIA fiber in each section, and arrowheads point toward fibers presenting TAs. Bar 50 μm.

The tibialis anterior of C57BL/6J adult mice is a fast contracting muscle, which, according to published studies [17,18,19] is composed of about 5% type-IIA, 35% type-IIX, and 60% type-IIB fibers, with some discrepancies depending on the age analyzed and the assay used to detect them. Tibialis anterior muscle has a superficial region with mainly type-IIB fibers, and a more profound region rich in IIX and IIA fibers [20]. To avoid sampling errors, we analyzed the MyHCs expression over the entire section of each muscle. As reported in Figure 1A (Figure 2 shows selected images for immunohistochemistry detection of MyHCs), the tibialis anterior of eight-months-old mice presented 63.70% ± 2.17 of type-IIB, 36.42% ± 3.46 of type-IIX, and 13.07% ± 1.17 of type-IIA fibers, while type-I fibers were almost not detectable.

In 18-month-old tibialis anterior, we counted 65.26% ± 2.40 of type-IIB, 36.23% ± 2.03 type-IIX, and 8.92% ± 1.05 type-IIA fibers, and in the oldest age group (24 months), we counted 75.84% ± 1.82 type-IIB, 28.19% ± 0.94 type-IIX, and 11.04% ± 2.74 type-IIA fibers. Therefore, age progression entails a significant increase of IIB fibers, to the detriment of IIX fibers, which show the tendency to decrease (*p* = 0.082) in very old samples, while type-IIA fibers’ percentage do not show major changes. 

The analysis of hybrid fibers suggests (Figure 1B) that the number of pure IIX decreases with age, and in turn, the number of hybrid IIA + IIX and IIB + IIX increases, leading us to conclude that, at these conditions, type-IIX fibers are the most negatively affected population in terms of presence.

### 2.2. Fiber-Type CSA in Aging Tibialis Anterior Muscle 

We measured the CSA of type-IIA, type-IIB, and type-IIX pure fibers. As reported in Figure 1C, IIB fibers display the largest CSA, IIX the intermediate, and IIA the smallest. In the considered age interval, no major modifications occur to the CSA of the three types of fiber. 

### 2.3. Fiber Type and Measurement of SDH Activity 

**Figure 3:**
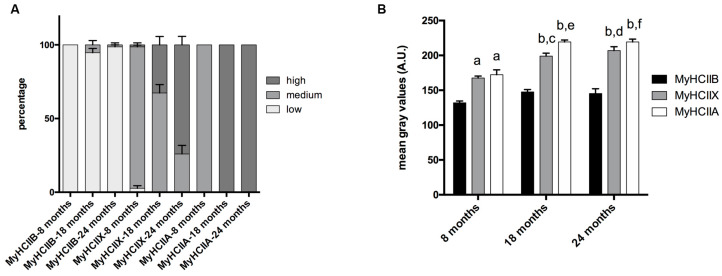
Fiber-type-dependent SDH activity during age progression. (**A**) The qualitative analysis indicates that type-IIB fibers display no difference in the three age groups, and type-IIX and -IIA fibers switch from medium activity to high activity during age progression. (**B**) The quantitative analysis of SDH activity indicates differences depending on fiber type and age (a, significantly different from age matched type-IIB fibers, *p* < 0.001; b, significantly different from age-matched type-IIB fibers, *p* < 0.0001; c, significantly different from eight-month type-IIX fibers, *p* < 0.01; d, significantly different from eight-month type-IIX fibers, *p* < 0.001; e, significantly different from eight month-old type-IIA fibers, *p* < 0.0001; f, significantly different from eight-month-old type-IIA fibers, *p* < 0.0001). In the *Y*-axis, the mean gray values in arbitrary units (A.U.) are reported. Data are expressed as means ± SEM.

The evaluation of the SDH assay reported in Figure 2, was performed visually, by dividing the muscle fibers into three groups, named high, medium, and low, based on the intensity of their SDH staining. The graph in Figure 3A reports the mean percentage of each intensity group in type-IIA, -IIX, and -IIB fibers. As shown in Figure 2 and Figure 3A, IIB fibers always present a low-intensity SDH staining that does not change during age progression. Type-IIX fibers display a medium-intensity staining that increasingly changes toward a very intense staining in old tibialis anterior. Type-IIA fibers from young mice display a medium intensity staining, but they rapidly switch toward a very intense staining from the middle-age phase onward. As a confirmation of the above-reported data, a quantitative analysis on a restricted group of 10 fibers per each fiber type was performed. Data on Figure 3B confirms that type-IIB fibers display the lowest SDH activity at different age-points, and IIX and IIA fibers exhibit an intermediate activity in young samples that increases with age. In agreement with the visual analysis, the quantitative analysis suggests that the increase in SDH activity occurs mainly in the 8–18-month time window, which corresponds to the transition from adult to middle age [15,16]. 

### 2.4. Capillarization 

**Figure 4:**
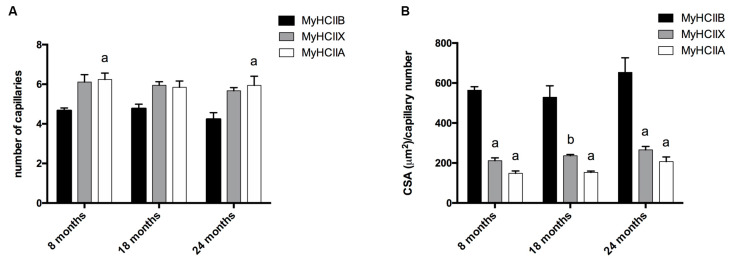
Analysis of fiber-type-dependent capillarization. (**A**) The capillary number depends on the fiber type (a, significantly different from age-matched type-IIB fibers, *p* < 0.05). (**B**) The analysis of the ratio between CSA and capillaries number indicates differences depending on fiber type (a, significantly different from age-matched type-IIB fibers, *p* < 0.0001; b, significantly different from age-matched type-IIB fibers, *p* < 0.001). Data are reported as means ± SEM.

We detected the capillaries in the tibialis anterior sections by means of alkaline phosphatase staining, as reported in Figure 2. The analysis of the level of capillarization in Figure 4A shows that the number of capillaries surrounding one fiber is dependent on the fiber type, with type-IIB fibers presenting fewer capillaries than IIX and IIA fibers. Moreover, the ratio of CSA to capillaries (Figure 4B), which provides an idea of the area served by a capillary, significantly decreases, going from IIB to IIX and IIA fibers. As a result of the increase of type-IIB fibers during age, the global capillarization level of the muscle decreases. 

### 2.5. Tubular Aggregates

**Figure 5:**
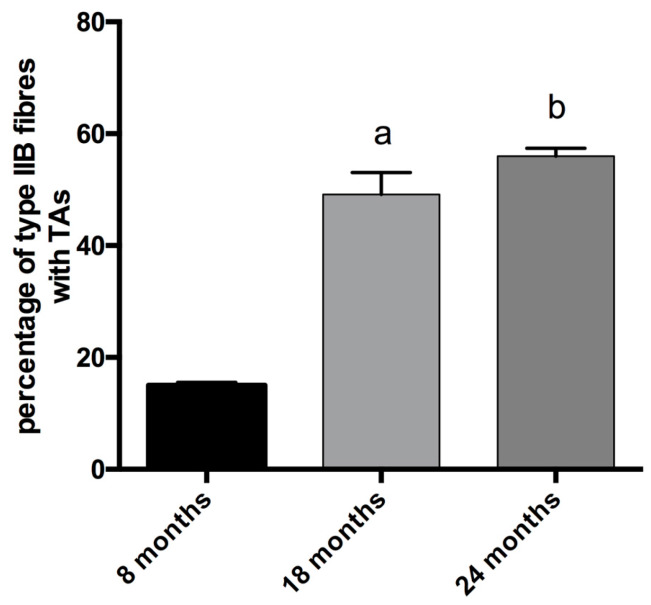
Analysis of the presence of TAs in type-IIB fibers. The percentage of IIB fibers with TAs increases with age (a, significantly different from eight-month-old mice, *p* < 0.001; b significantly different from eight-month-old mice, *p* < 0.0001). Data are reported as means ± SEM.

In physiologically aging C57BL/6J male mice, TAs develop in fibers expressing MyHC IIB [21]. Therefore, we counted the number of TAs in type-IIB fibers from tibialis anterior muscles at different ages (see Figure 2), and we observed that type-IIB fibers from eight-month-old mice presented few TAs; meanwhile, the number of TAs in type-IIB fibres was significantly raised in 18- and 24- month-old animals, with the most important increase taking place in the age interval of 8–18 months, as shown in Figure 2 and Figure 5.

## 3. Discussion

In the present study, we performed the analysis of the fiber profile of the tibialis anterior muscle of physiologically aging C57BL/6J mice, by investigating the expression of the MyHCs, the capillarization, the Succinate Dehydrogenase (SDH) activity, and the presence of tubular aggregates (TAs). The present data confirm that the tibialis anterior is a fast-contracting muscle that, with age progression, exhibits significantly more type-IIB fibers to the detriment of type-IIX fibers that display a negative trend, whereas type-IIA fibers seem not to undergo major quantitative changes. Interestingly, a similar fiber-type switch was observed in the oldest-old humans with a low degree of motility [8], due to disuse atrophy that results in a slow-to-fast fiber transition [3,8]

Interestingly, the CSA of the three different fiber types do not show major variation in the age interval analyzed. Although rodent muscles, different from human muscles, express MyHCIIB, these data are in partial agreement with recent data showing that age is accompanied by an increase of fast-contracting fibers, but it does not dramatically affect fiber size in oldest-old individuals with different degrees of mobility [8,14].

Aiming to gather further information on the mechanisms contributing to the variation of the functional properties during skeletal muscle aging [22,23], we analyzed multiple histological parameters, to build the fiber profile of tibialis anterior during aging. In physiological conditions, the MyHCs isoform’s expression in a muscle fiber determines its contractile properties and is associated with its metabolic properties [2,3]. However, the recent data evidencing that the different fibers can display different mitochondrial activity and capillarization depending on physical activity, and that their metabolic profile muscle fibers can vary without the change of the MyHCs isoforms expression [4], oriented our work toward more complete analyses of the fiber profile. 

The SDH staining of sections from different age-points shows that type-IIX and -IIA fibers, which are more oxidative than IIB fibers, progressively shift toward an even more oxidative profile in aged muscles, suggesting that, during aging, the metabolic profile of a fiber can change. These data are in agreement with recent evidences showing that, during aging, human muscle fibers can change the pathway used to produce ATP [24,25].

Despite the presence of contradictory results in the literature [26], it has been suggested that muscle performance may also depend on the capillarization [5,27,28], since the blood flow provides oxygen for muscle contraction, glucose, and nutrients and moreover helps the removal of waste product, with the final aim of maintaining a correct metabolic equilibrium in the muscle fiber. Accordingly, it has been proposed that capillary rarefaction may contribute to the age-related decline of muscle function, making capillarization one of the determinants of a lower exercise capacity in older adults [10,29,30]. The analysis of the level of capillarization in aging C57BL/6J mice indicates that the number of capillaries surrounding one fiber is dependent on the fiber type, with type-IIB fibers presenting fewer capillaries than IIX and IIA fibers. Moreover, the ratio CSA:capillaries, which provides the area served by a capillary, significantly decreases going from glycolytic to oxidative fibers. The increase in type IIB and the increasing trend of the CSA:capillaries in aged animals ratio suggest a global loss of oxygenation of the tibialis anterior muscle. 

Interestingly, data on capillarization can seem contradictory with the evidence that SDH activity increases in type IIX and IIA of older animals. It has been reported that human muscle fibers can change the pathway to produce ATP needed for the contraction during aging [24,25]. However, it has also been suggested that the capillarization has a predominant role, since it determines oxygen diffusion in muscle fibers [27]. The uncoupling between cell metabolisms and oxygen supply could be a trigger in the alteration of the contractile properties of a muscle.

Tubular Aggregates are intracellular structures that develop in type-IIB fibers of selected mice strains, depending on sex, age, or muscle pathologies [12,13,21], due to a defective mitochondrial activity, and to the accumulation of oxidative stress by-products in the myofibers [11,12]. Our data show that aging entails a significant increase of TAs in type-IIB fibers. Together with the evidence that the tibialis anterior undergoes a global loss of capillarization is worth to think that with age progression the cell equilibrium is compromised and TAs’ onset is favored. Recent data from the laboratory [31] show an inverse correlation between the number of capillaries and the presence of TAs, suggesting that the reduction of capillarization of the muscle tissue contributes to the formation of TAs. Moreover, we recently showed that the supplementation of Resveratrol in the diet improved the mitochondrial activity and the inflammatory condition, reduced the number of TAs in type-IIB fibers, and induced the improvement of the fatigue resistance in an ex vivo test [32]. It is conceivable that the generalized reduction of capillarization, which is mainly attributable to the increase of type-IIB fibers, together with the increase of the mitochondrial activity of type-IIA and -IIX fibers, can be one of the determinants of the development of TAs. In fact, the continuous contraction of the skeletal muscle cell requires the corresponding maintenance of the cell conditions (i.e., pH, reactive oxygen species). This is facilitated when the skeletal muscle has an appropriate capillary network to provide nutrients and remove waste products from the muscle cell. Thus, the loss of capillarization can result in a redox unbalance and, as previously suggested [11,12], induce the formation of TAs. 

As a result, the presence of TAs in aged muscles can cause an alteration of the contraction parameters. Interestingly, EDL, a muscle similar to the tibialis anterior for the fiber-type composition, does not present major changes in the contraction time, half-relaxation time, or force–velocity relationship during aging, while it displays a significantly modified tetanus [23]. The presence of TAs could have a role by reducing the space occupied by the myofibrils in a fiber, alter the sarcoplasmic reticulum functionality [9], and compromise contractile properties.

Although our data confirm that mice skeletal muscles in the reported age range do not undergo dramatic changes [6], they partially mirror the muscle atrophy observed in humans, as evidenced by the increase fast fibers and the maintenance of CSA. Moreover, our work detected the increase of SDH activity in a fiber-type specific manner and the onset of TAs in type-IIB fibers. These evidences, together with data from the literature, suggest that the decline in skeletal muscle performance does not depend on a single, but on multiple, factors [9,33,34]. Considering that muscle contraction is achieved thanks to the orchestration of the heterogeneity of muscle fibers, it is conceivable that the small modifications, observed at multiple levels, contribute to the alteration of the contractile properties. 

This complex combination of effects makes age-related muscle atrophy a difficult therapeutic problem, where interventions on multiple or broad molecular targets, that can be modulated by caloric restriction or antioxidants [6], could in principle be successful in the treatment of skeletal muscle aging. The emerging application of natural molecules in the treatment of muscle aging should be considered in this perspective, as for example, the administration of Resveratrol, a potent natural antioxidant described for its ability to interact with multiple cell pathways [35], and which has been shown to improve the histological characteristics of the aging skeletal muscles and the resistance to fatigue in aging mice, both in ex vivo and in vivo experiments [32,36]. 

## 4. Materials and Methods 

### 4.1. Animals 

C57BL/6J male mice aged 8, 18, and 24–28 months [15,16] were kept in the local animal house, in accordance with the European legislation on the use and care of laboratory animals (EU Directive 2010/63) and were approved by the Ethics Committee of the University of Siena and from Ministero della Salute, Italy (Project n° J-21/10/10; 21^st^ October 2010). At the reported ages, the animals were sacrificed, and the tibialis anterior muscles were harvested for histological analyses.

### 4.2. Antibodies

In the immunohistochemistry experiments, the following primary antibodies were utilized: anti MyHC IIB, clone BF-F3 (Developmental Studies Hybridoma Bank, Iowa City, Iowa, USA), at 4 μg/mL; anti MyHC IIX/D, clone 6H1 (Developmental Studies Hybridoma Bank, Iowa City, Iowa, USA), at 4 μg/mL; anti MyHC IIA, clone SC-71 (Developmental Studies Hybridoma Bank, Iowa City, Iowa, USA), at 4 μg/mL; and anti Myosin-Slow, clone NOQ7.5.4D (Sigma Aldrich, Milano, Italy), diluted 1:500. The anti-mouse biotinylated secondary antibody raised in horse (Vector Laboratories, Burlingame, CA USA) was used at the dilution of 1:200. 

### 4.3. Cryostat Sectioning 

Tibialis anterior muscles were dissected from mice, directly frozen in isopentane cooled in liquid nitrogen, and cryoprotected with Tissue-Tek II OCT compound (Sakura Finetek USA, Inc., Torrance, CA, USA). Transverse serial sections, 8 μm thick, were cut with a Leica cryostat (CM 1850, Leica Microsystem, Wetzlar, Germany). 

### 4.4. Immunohistochemistry Reactions 

Air-dried sections were blocked with 5% normal horse serum in PBS, to avoid non-specific binding of the antibodies, and incubated with primary antibodies in a humidified chamber, at room temperature, for 2 h. Specimens were washed three times with PBS and then incubated 1 h with an anti-mouse biotinylated secondary antibody raised in horse, washed, and incubated with VECTASTAIN ABC Reagent (Vector Laboratories, Burlingame, CA USA) for 30 min. After washing, the sections were incubated in Diamino Benzidine solution (SigmaFastDAB; Sigma Aldrich, Milano, Italy), until development of staining. Sections were dipped in water, dehydrated with 95% and absolute ethanol alcohol, followed by clarification in BioClear (BioOptica, Milano, Italy), and mounted with a resinous medium. Images were acquired with a Leica Orthoplan microscope (Leica, Wetzlar, Germany) equipped with a Leica DC-100 digital camera (Leica, Wetzlar, Germany). 

### 4.5. SDH Staining 

Frozen sections were directly placed from −80 °C in freshly prepared SDH solution (Natrium Succinate 0.2M, Nitro Blue Tetrazolium (Sigma Aldrich, Milano, Italy) 1 mg/mL in Tris-HCl buffer 0.2M, pH 7.4), and incubated 15–20 min. The enzymatic reaction was stopped by dipping the slides in water, which were subsequently air-dried in the dark and mounted in gelatin-glycerol. Images were acquired with a Leica Orthoplan microscope (Leica, Wetzlar, Germany) equipped with a Leica DC-100 digital camera (Leica, Wetzlar, Germany). For the visual SDH quantitation, muscle fibers were allocated in three groups, depending on the intensity of the staining: high, medium, and low. For quantitative analyses, specimens acquisition was performed with a multi-position bright-field microscope (Scan R; Olympus, Shinjuku, Tokyo, Japan), SDH activity was calculated on 10 fibers (per animal and per different fiber type), with Fiji free software [37], by converting images to grayscale and measuring the mean gray intensity values [32]. 

### 4.6. Alkaline Phosphatase Staining 

Capillaries were analyzed by alkaline phosphatase staining, as previously described [38]. Air-dried sections were incubated in a freshly prepared 50 mM Tris-HCl pH 9.5 solution containing 0.14 mg/mL Nitro Blue Tetrazolium (Sigma Aldrich, Milano, Italy), 0.07 mg/mL 5-bromo-4-chloro-3-indolylphosphate (BCIP, Sigma Aldrich, Milano, Italy), and 2.3 mM Magnesium Chloride, for 15 min, at 37 °C. Sections were extensively washed in water, and counterstained in 0.5% eosin, washed in water, dehydrated with 95% and absolute ethanol alcohol, followed by clarification in BioClear clearing reagent (BioOptica, Milano, Italy), and mounted with a resinous medium. Images were acquired with a Leica Orthoplan microscope (Leica, Wetzlar, Germany) equipped with a Leica DC-100 digital camera (Leica, Wetzlar, Germany). Images were processed with Fiji free software [37], and the CSA of the fibers was determined by carefully drawing a polygon around each fiber; the number of capillaries per fiber was determined by counting the alkaline phosphatase–positive spots surrounding each fiber. 

### 4.7. Toluidine Blue Staining

Tibialis anterior transversal sections were incubated for 2 min in a 0.1% Toluidine Blue solution, dipped in water, dehydrated in ethanol, clarified in BioClear clearing reagent (BioOptica, Milano, Italy), and mounted with a resinous medium. Images were acquired with a Leica Orthoplan microscope (Leica, Wetzlar, Germany) equipped with a Leica DC-100 digital camera (Leica, Wetzlar, Germany).

### 4.8. Statistical Analyses

The fiber-type determination was performed by analyzing the entire section of tibialis anterior muscle of 3 mice per age group. A range of 2500–4000 fibers was counted for each animal. For CSA determination, capillaries number, SDH assay, and 100 to 200 fibers per animal, depending on their abundance, were analyzed. The percentage of type-IIB fibers presenting TAs was determined on 150–250 fibers per animal.

Significant differences among different age groups were determined with GraphPad Prism version 7.00 for Windows, GraphPad Software, La Jolla, California, USA (www.graphpad.com), by performing one way ANOVA analyses. Data are expressed as means ± SEM.
